# Immediate dental implants placed into infected sites present a higher risk of failure than immediate dental implants placed into non-infected sites: Systematic review and meta-analysis

**DOI:** 10.4317/medoral.22954

**Published:** 2019-06-25

**Authors:** Olavo-Barbosa de Oliveira-Neto, Cleidiel-Aparecido-Araújo Lemos, Fabiano-Timbó Barbosa, Célio-Fernando de Sousa-Rodrigues, Fernando-José Camello de Lima

**Affiliations:** 1 DDS, MSc student, Department of Morphology, Anatomy Area, Piracicaba Dental School, University of Campinas, Piracicaba, São Paulo, Brazil; 2 DDS, MSc, PhD student, Department of Prosthodontics, Araçatuba Dental School, São Paulo State University (UNESP), Araçatuba, São Paulo, Brazil; 3 MD, PhD, Professor, Medical School, Federal University of Alagoas (UFAL), Maceió, Alagoas, Brazil; 4 MD, PhD, Professor, Department of Morphology, Human Anatomy Area, Institute of Health and Biological Sciences, Federal University of Alagoas (UFAL), Maceió, Alagoas, Brazil; 5 DDS, PhD, Professor, Department of Morphology, Human Anatomy Area, Institute of Health and Biological Sciences, Federal University of Alagoas (UFAL), Maceió, Alagoas, Brazil

## Abstract

**Background:**

Alveolar infection is known as a risk factor for implant failure. Current meta-analysis on the theme could not prove statistically that immediate dental implants placed into infected sites have a higher risk of failure than immediate dental implants placed into non-infected sites. The purpose of this meta-analysis was to determine the effectiveness of immediate dental implants placed into infected versus non-infected sites.

**Material and Methods:**

Seven databases were sought by two reviewers. Randomized or non-randomized clinical trials that compared the placement of dental implants into infected versus non-infected sites were eligible for the study. Exclusion criteria were: papers in which the survival rate was not the primary outcome; papers without a control group; studies with less than one year of follow-up; studies whose patients did not receive antibiotic therapy; studies with medically compromised patients; duplicated papers. Risk of bias assessment was performed with the Cochrane Collaboration tool.

**Results:**

Of the 3.253 initial hits, 8 studies were included in both qualitative and quantitative synthesis (kappa=0.90; very good agreement). Forest plot for implant failure showed that immediate implants placed into infected sites presented a statistically significant risk of failure that is almost 3 times higher than when placed into non-infected sites (risk ratio= 2.99; 95% confidence interval: 1.04, 8.56; *p*= 0.04; 935 implants; i2= 0%). Peri-implant outcomes showed no statistical difference.

**Conclusions:**

Immediate dental implants placed into infected sites presented a statistically significant higher risk of failure than immediate dental implants placed into non-infected sites. Peri-implant outcomes were not statistically affected in this intervention.

** Key words:**Dental implants, infection, tooth socket, systematic review, immediate placement.

## Introduction

The placement of immediate dental implants into infected sites is known in implant dentistry as a potential risk factor for implant failure. However, patients and practitioners started to realize that the number of treatment sessions could be reduced from the time of dental extraction to implant placement, which would provide a reduction of treatment costs and accelerate the treatment process. Surgeons also advocate that a larger width of peri-implant keratinized mucosa is maintained and that there is a guarantee of bone presence in the time of surgery when immediate implants are used, which may be reduced if the surgeon chooses to wait a healing period up to 4 or 6 months (1,2).

In order to address this controversy, primary studies regarding this important topic were initially conducted using dogs as sample on the late 1990’s and early 2000’s (3,4). Then, primary studies with humans were conducted, and the first systematic reviews were then performed with no solid evidence and mixed results from animals and humans (5). Meta-analysis on this theme are quite recent and could not prove true the recommendation to avoid immediate implants in infected sites, which increases the controversy surrounding this particular theme (1,6).

Despite systematic reviews exist on this topic, they were recently assessed in a tertiary study, which showed that their results and conclusions are not reliable (1,6,7,9-11) .In addition, there is no current statistical prove that the survival rate of immediate dental implants placed into infected sites is affected by this condition. Thus, the present systematic review was conducted to answer the following focused question: what is the effectiveness of immediate dental implants placed into infected versus non-infected sites?

## Material and Methods

This systematic review was reported in accordance to the Preferred Reporting Items for Systematic Reviews and Meta-Analysis Protocols (PRISMA-P) and followed the recommendations of the Cochrane Collaboration Handbook for Systematic Reviews of Interventions (12,13). A protocol was developed a priori and is available for consultation at the International Prospective Register of Systematic Reviews (PROSPERO - http://www.crd.york.ac.uk/PROSPERO) with the registration number CRD42018092156.

Seven online databases (PubMed, Embase, Scopus, Web of Science, CENTRAL, LILACS, and Open Grey) were sought for eligible studies from database inception to May 2018. In order to perform a comprehensive online search, a search string algorithm was generated specifically for each database as follows:

- PubMed (by MeSH): (((((((((((dental implants) OR implants, dental) OR dental implant) OR implant, dental) OR dental prostheses, surgical) OR dental prosthesis, surgical) OR surgical dental prosthesis) OR surgical dental prosthesis) OR prostheses, surgical dental) OR prosthesis, surgical dental)) AND ((((((((immediate) OR immediately) OR immediate placement) OR immediately placed)) OR placement) OR dental implant placement) OR oral implant placement) AND (((((((((((((((((((infected) OR infection) OR infected sites) OR infected socket) OR periapical lesion) OR periodontal lesion) OR endodontic lesion)) OR periodontitis))))) OR infection control, dental) OR dental infection control) OR control, dental infection) OR controls, dental infection) OR dental infection controls) OR infection controls, dental)

- Embase (by EmTree): (‘infection’/exp OR ‘acute infection’ OR ‘chronic infection’ OR ‘focal infection’ OR ‘infection’ OR ‘infection, focal’ OR ‘tooth infection’/exp OR ‘dental infection’ OR ‘focal infection, dental’ OR ‘infection, dental’ OR ‘odontogenic infection’ OR ‘tooth infection’ OR ‘infected sites’ OR ‘infected socket’ OR ‘periapical lesion’ OR ‘periodontal lesion’ OR ‘endodontic lesion’ OR ‘periodontitis’/exp) AND (‘tooth implantation’/exp OR ‘dental implantation’ OR ‘dental implantation, endosseous’ OR ‘dental implantation, endosseous, endodontic’ OR ‘dental implantation, subperiosteal’ OR ‘tooth implantation’ OR ‘tooth implant’/exp OR ‘dental implant’ OR ‘dental implants’ OR ‘endosseous dental implant’ OR ‘implant, teeth’ OR ‘implant, tooth’ OR ‘implants, teeth’ OR ‘implants, tooth’ OR ‘teeth implant’ OR ‘teeth implants’ OR ‘tooth implant’ OR ‘tooth implants’ OR ‘dental prostheses, surgical’ OR ‘dental prosthesis, surgical’ OR ‘surgical dental prostheses’ OR ‘surgical dental prosthesis’ OR ‘prostheses, surgical dental’ OR ‘prosthesis, surgical dental’) AND (immediate OR immediately OR ‘immediate placement’ OR ‘immediately placed’ OR placement OR ‘dental implant placement’ OR ‘oral implant placement’)

- Scopus:

#1 - TITLE-ABS-KEY ( “immediate placement” OR “immediately placed” )

#2 - TITLE-ABS-KEY ( “dental implant” OR “dental implants” OR “tooth implant” )

#1 AND #2: TITLE-ABS-KEY ( “immediate placement” OR “immediately placed” ) AND TITLE-ABS-KEY ( “dental implant” OR “dental implants” OR “tooth implant” )

- Web of Science:

#1 - ts=(immediate placement OR immediately placed)

#2 - ts=(dental implant OR dental implants OR tooth implant)

#3 - ts=(infected sites OR infected sockets OR infected OR infection)

#1 AND #2 AND #3

- CENTRAL:

#1 - MeSH descriptor: [Dental Implants] explode all trees

#2 - immediate placement:ti,ab,kw (Word variations have been searched)

#3 - immediately placed:ti,ab,kw (Word variations have been searched)

#4 - infected sites:ti,ab,kw (Word variations have been searched)

#5 - infected sockets:ti,ab,kw (Word variations have been searched)

#6 - #1 and #2 or #3 and #4 or #5

- LILACS:

#1 = “dental implant” OR “tooth implant” OR “tooth implantation”

#2 = “immediate placement” OR “immediately placed”

#1 AND #2

- Open Grey:

#1 = “immediate placement” OR “immediately placed”

#2 = “dental implant” OR “dental implants” OR “tooth implant” OR “tooth implantation”

#1 AND #2 = “immediate placement” OR “immediately placed” AND “dental implant” OR “dental implants” OR “tooth implant” OR “tooth implantation”

Online searches were conducted at the Federal University of Alagoas and at São Paulo State University by two independent reviewers (O.B.O.N. and C.A.A.L.). Initial hits were sought through title and/or abstract reading. Potential eligible studies were then selected and fully read. Final decision would rely on the mutual agreement between theses reviewers that a study should be included. In cases were a disagreement occurred, a third and more experienced reviewer (F.J.C.L.) would be consulted to break the tie (14). Reference lists of included publications were also checked for additional records. Search and selection processes did not set restrictions of language or type of publications. Corresponding authors of included papers would be consulted via e-mail if there was a need to clarify the report of theirs studies.

Randomized or non-randomized clinical trials that compared the placement of immediate dental implants into infected and non-infected sites were included. Exclusion criteria were: papers in which the survival rate was not the primary outcome; papers which did not have a control group (non-infected sites); studies with less than 1 year of follow-up; studies in which patients did not receive antibiotic therapy; studies that included medically compromised patients; and duplicated publications.

The primary outcome was the survival rate of dental implants; secondary outcomes were: peri-implant bone loss; plaque index; bleeding index; probing depth; and width of peri-implant keratinized mucosa. Complementary outcomes were: follow-up period; number of patients; number of implants; and patient’s age range.

Risk of bias assessment

Clinical trials were assessed with the Cochrane Collaboration’s tool for risk of bias assessment. This tool features the following items: a) random sequence generation; b) allocation concealment; c) blinding of participants and personnel; d) blinding of outcome assessment; e) incomplete outcome data; f) selective reporting; g) other bias. Possible answers for each item were “low risk of bias”, “unclear risk of bias”, and “high risk of bias” and were graphically represented, respectively, as green, yellow, or red colors, as in a traffic light system (12).

One reviewer (O.B.O.N.) performed the assessment and a second reviewer (F.J.C.L.) checked the first reviewer’s assessment. A consensus was established for all items; therefore, it was not necessary to consult a third reviewer (F.T.B.).

-Data analysis

It was not necessary to perform a sample size calculation since the present study is a systematic review. Cohen’s kappa statistics was performed to measure the level of agreement between reviewers on the selection of eligible studies and risk of bias assessment.

The survival rate of dental implants, plaque index, and bleeding index were described as percentages; peri-implant bone loss, probing depth, and width of peri-implant keratinized mucosa were described in millimeters. Complementary outcomes were described as in the reports of original studies.

The risk ratio (RR) estimative was calculated for dichotomous outcomes and the mean difference (MD) was calculated for continuous outcomes. The random effects model was used, and the confidence interval was set at 95%.

Heterogeneity between studies was calculated using a Chi-squared test and estimated by the Higgins Test (I2 statistics), whereas it would be considered significant with a p-value inferior to 10% (*p*<0.10) and an I2 result higher than 50% was considered as substantial heterogeneity. Sensitivity analysis was planned and would include the comparison of studies of the same type, the exclusion of the analysis of studies with high risk of bias, and by reanalyzing data through variation of missing data. Funnel plot analysis of publication bias would be performed if the outcome was reported in a minimum number of 10 studies. Statistical analysis was conducted on the software Review Manager 5.3.

Additionally, a weighted mean was calculated on Microsoft Excel considering the secondary outcomes and the follow-up period in order to provide the information of how the outcomes behave in different periods of follow-ups. A dental implant was considered as the statistical unit to perform all analysis.

## Results

**Figure 1:**
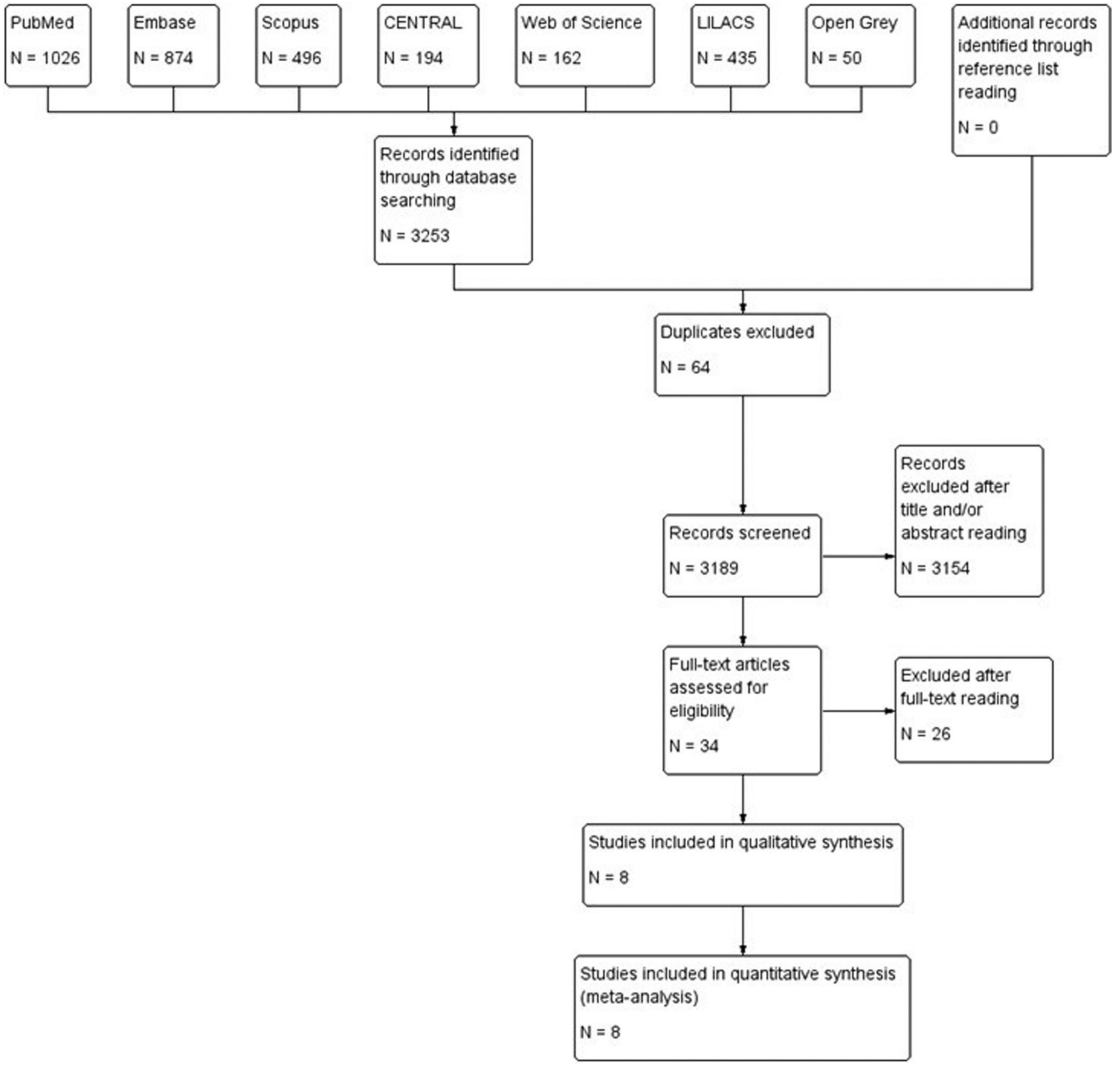
Flow chart showing steps performed to select eligible studies for systematic review.

Online searches yielded a total of 3.253 initial hits considering all databases, as follows: 1.026 on PubMed, 874 on Embase, 496 on Scopus, 162 on Web of Science, 435 on LILACS, 194 on Cochrane CENTRAL, and 50 on Open Grey. After the exclusion of 64 duplicated papers, 3.154 records were excluded trough title and/or abstract reading. Then, 34 full-text articles were assessed for eligibility and 26 papers were excluded for the reasons listed on  Table 1 (15-40). Finally, 8 studies were considered for both qualitative and quantitative synthesis (kappa=0.90; very good agreement) (41-48). Figure 1 summarizes all steps performed on the present systematic review. Characteristics of included studies are summarized on  Table 2.

**Table 1:**
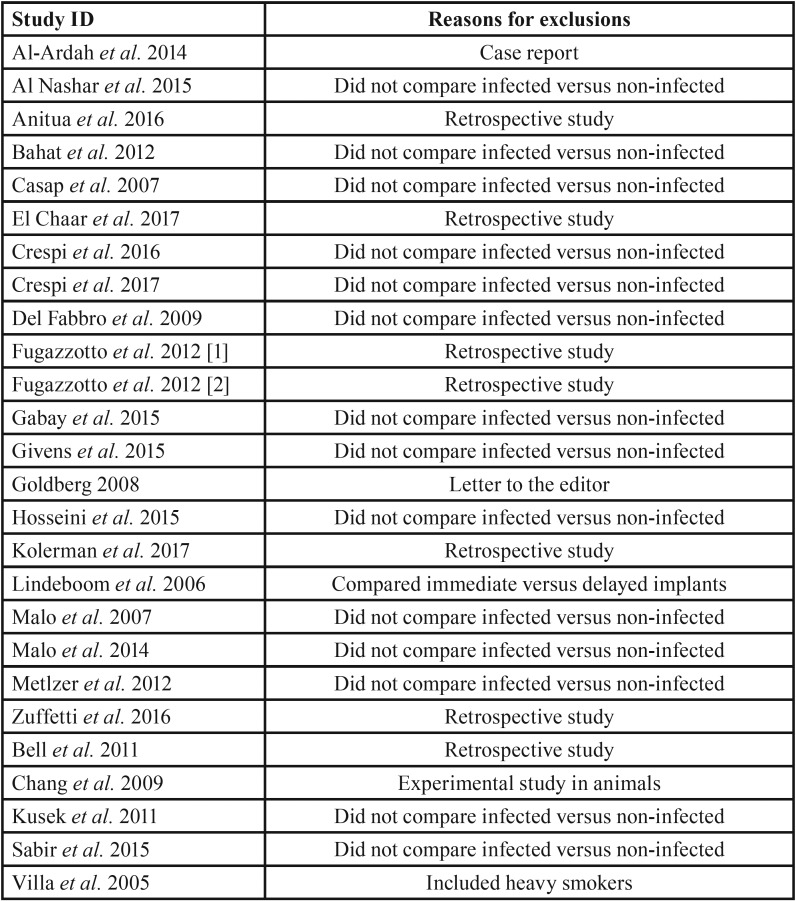
List of fully read excluded studies and reasons for exclusions.

**Table 2:**
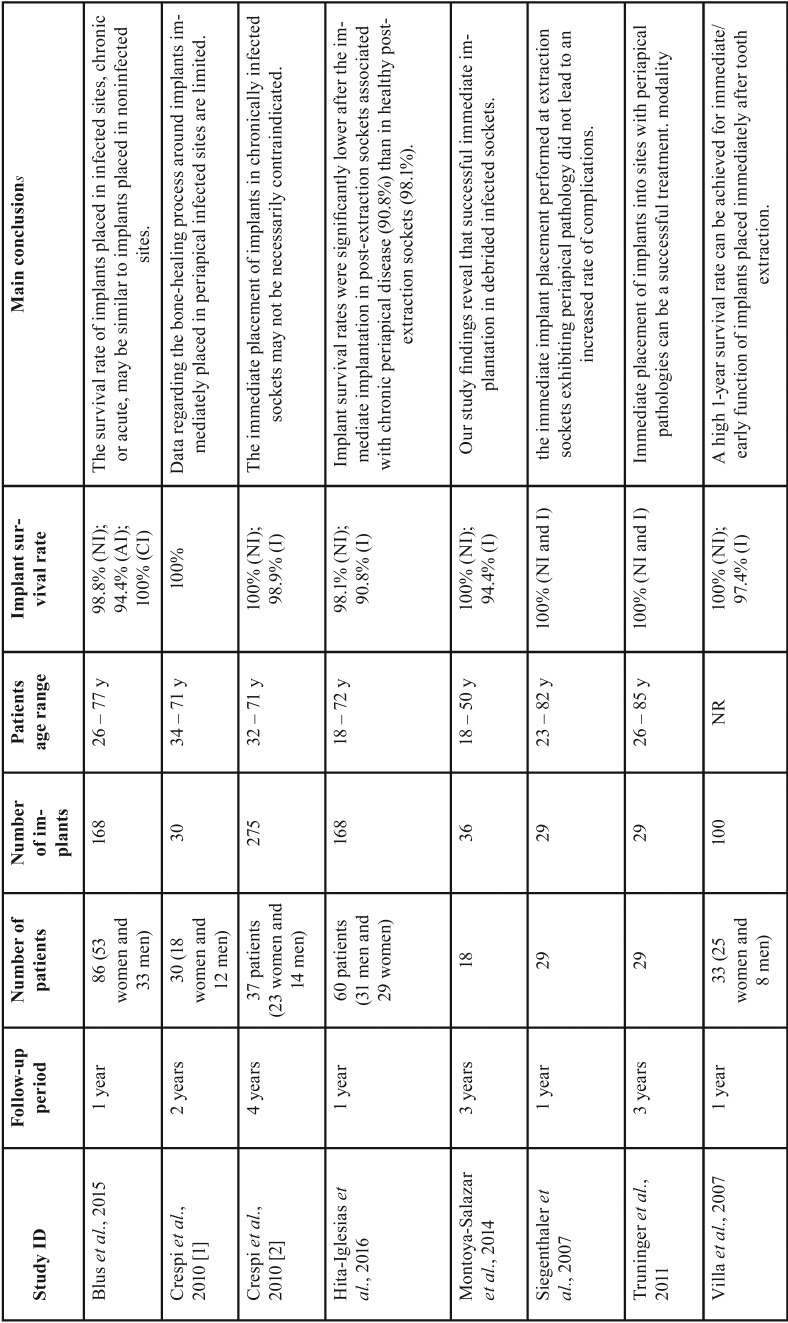
Characteristics of included studies. NI=non-infected; I=infected; AI=acute infected; CI=chronically infected; NR=not reported.

**Figure 2:**
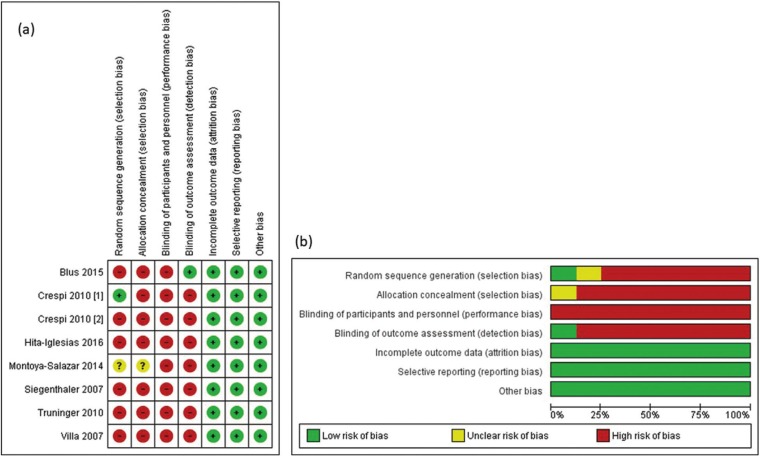
Risk of bias summary (a) and risk of bias graph (b) of included studies using the Cochrane Collaboration tool.

Risk of bias assessment showed that included studies were of unclear risk of bias, whereas all papers presented low risk of bias for the items “incomplete outcome data”, “selective reporting”, and other bias; however, the item “blinding of participants and personnel” presented high risk of bias in all included studies. The remaining items (“random sequence generation” and “allocation concealment”) were mostly assessed as of high risk of bias. Figure 2 shows risk of bias summary and risk of bias graph of eligible papers. Cohen’s kappa statistics showed an inter-reviewer agreement rate of 0.81 (strong agreement) for risk of bias assessment.

**Figure 3:**
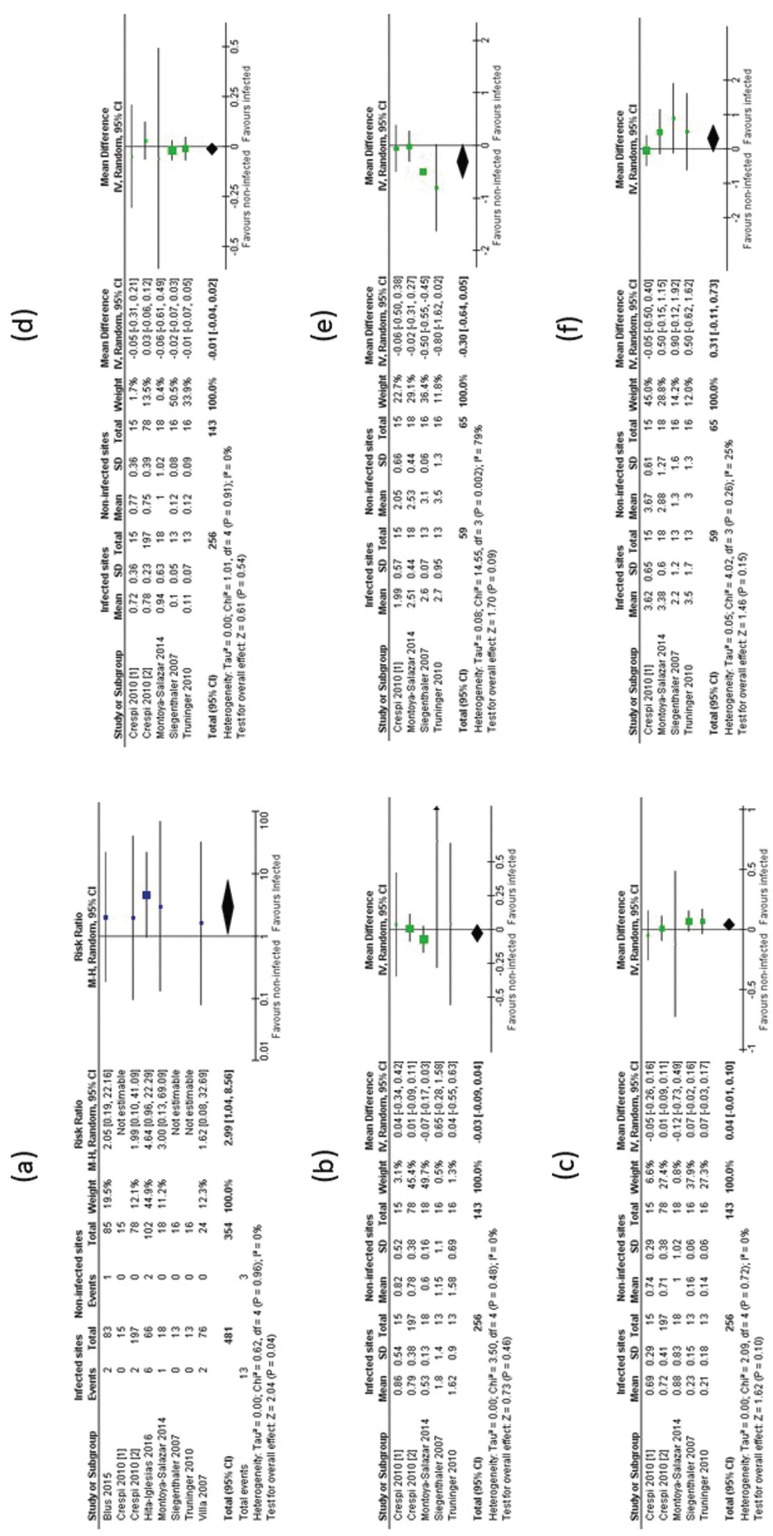
Forest plots for: implant failure (a); peri-implant bone loss (b); plaque index (c); bleeding index (d); probing depth (e); width of peri-implant keratinized mucosa (f). Implant failure exhibited statistical significant difference for the meta-analysis.

Implant survival rate: the eight included studies reported this outcome, which ranged from 90.8% 100.0% (41-48). Meta-analysis was performed for implant failure, which showed statistically significant difference (risk ratio = 2.99; 95% confidence interval: 1.04, 8.56; *p*= 0.04; 935 implants; i2= 0%). This indicates that immediate dental implants placed into infected sites present a risk of failure that is almost 3 times higher than immediate implants placed into non-infected sites. Figure 3(a) shows the forest plot for implant failure.

Peri-implant bone loss: five studies reported this outcome and it did not present statistical difference on the meta-analysis (mean difference= -0.03; 95% confidence interval: -0.09, 0.04; *p*=0.46; 399 implants; i2= 0%). Figure 3(b) shows the forest plot for peri-implant bone loss (42,43,45-47).

Meta-analysis for additional peri-implant outcomes are shown of Figure 3(c) to (f): the outcomes bleeding index and plaque index were reported in 5 studies, totaling 399 assessed immediate dental implants and there were no statistical difference on meta-analysis, which presented, respectively, the following results: mean difference= 0.05; 95% confidence interval:-0.01, 0.10; *p*= 0.08; i2= 0%; and mean difference= -0.01; 95% confidence interval: -0.04, 0.02; *p*= 0.54; i2 = 0% (42,43,45-47).

The outcomes probing depth and width of peri-implant keratinized mucosa were reported in 4 studies, totaling 124 immediate dental implants assessed and also there were no statistically significant difference on meta-analysis, which presented, respectively, the following results: mean difference = -0.30; 95% confidence interval: -0.64, 0.05; *p*= 0.09; i2= 79%; and mean difference= 0.31; 95% confidence interval: -0.11, 0.73; *p*= 0.15; i2= 25%.

We also calculated weighted means considering the secondary (peri-implant) outcomes and the follow-up periods. The outcomes peri-implant bone loss, plaque index, and bleeding index were reported in five studies with 1, 2, 3, and 4 years of follow-up, with mean follow-up of 2.6 years (+/-1.1); the outcomes probing depth and width of peri-implant keratinized mucosa were described in 4 studies with 1, 2, and 3 years of follow-up, with mean follow-up of 2.25 years (+/-0.95). One must highlight the following results: the outcome peri-implant bone loss showed a higher mean loss of marginal bone per year of follow-up in infected groups 0.62 mm (+/-0.67) than in non-infected groups 0.49 mm (+/-0.39); and the outcome plaque index showed a higher accumulation per year of plaque in infected groups 0.62 (+/-0.67) than in non-infected groups 0.21 (+/-0.13). More detailed data for these outcomes are shown, respectively, on  Table 3 and  Table 4.

**Table 3:**
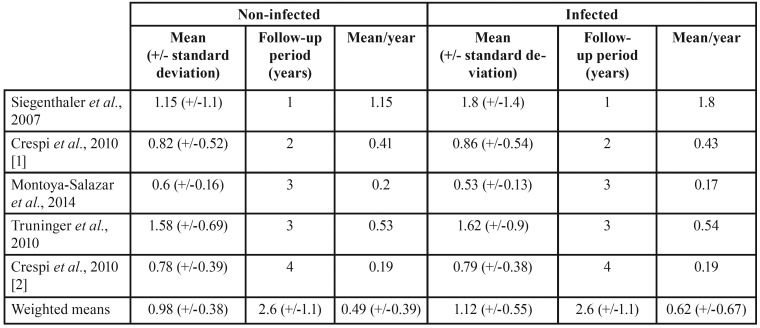
Peri-implant bone loss (in millimeters) as reported on primary studies and weighted means calculated from these data (original data from the present study).

**Table 4:**
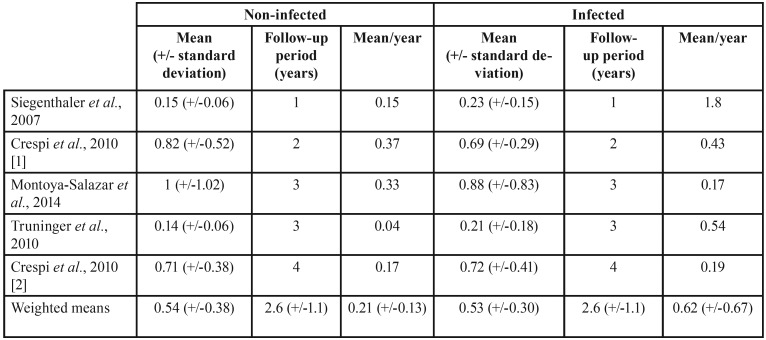
Plaque index (in fractional percentages) as reported on primary studies and weighted means calculated from these data (original data from the present study).

The remaining outcomes showed an equilibrium between non-infected and infected groups for the weighted means considering the reported values and the follow-up periods. The outcome bleeding index presented, in fractional percentages, 0.21 (+/-0.14) in non-infected groups and 0.2 mm (+/-0.13) in infected groups; the outcome probing depth presented for non-infected and infected groups, respectively, values of 1.53 mm (+/-1.05) and 1.33 mm (+/-0.84); and the outcome width of peri-implant keratinized mucosa exhibited values of 1.27 mm (+/-0.40) in non-infected groups and 1.57 mm (+/-0.52) in infected groups.

## Discussion

The present meta-analysis yielded the findings of 8 clinical trials regarding the placement of immediate dental implants into infected versus non-infected sites and showed that dental implants placed into infected sites have a risk of failure that is almost 3 times higher compared to non-infected sites, with statistically significant difference (41-48).

Meta-analyses for peri-implant outcomes (peri-implant bone loss, plaque index, bleeding index, probing depth, and width of keratinized mucosa) showed no statistically significant difference (42,43,45-47). The only outcome that exhibited substantial heterogeneity was probing depth (I2=79%); however, a funnel plot analysis was not conducted to assess publication bias for this outcome (and for all others) because of insufficient number of studies (12). In addition, sensitivity analysis was not performed because all included studies were of the same type, there were no missing data, and all studies were of unclear risk of bias.

Despite not showing statistical significant results on meta-analysis, it’s worth mentioning that results regarding the outcome values calculated per year of follow-up (original data from the present study) can be considered of clinical importance and deserve special attention, especially the outcomes peri-implant bone loss and plaque index, which showed, respectively, a higher mean loss of marginal bone loss per year of follow up in infected groups 0.62 mm (+/-0.67) than in non-infected groups 0.49 mm (+/-0.39), and a higher accumulation of plaque per year of follow up in infected groups 0.62 (+/-0.67) than in non-infected groups 0.21 (+/-0.13). These weighted means may be an indication that peri-implant tissues may show a higher rate of peri-implant mucositis around immediate dental implants placed into infected sites than in immediate dental implants placed into non-infected sites. However, considering the standard deviations presented, one may realize that the relative equivalence of these results shows that peri-implant tissues in both groups (infected and non-infected) behave the same, and therefore the real reason for an implant loss would rest on the fact that the dental socket was not properly disinfected prior to implant placement and not because of patient-related habits.

The present study features the following strengths: this is, to the best of the authors’ knowledge, the first meta-analysis that could prove statistically that immediate dental implants placed into infected sites present a higher risk of failure compared to non-infected sites, whose values were not only statistically significant but also clinically relevant; in addition, only clinical trials with humans were included, which substantially increases external validity (an important concern identified on the first systematic reviews on the theme, which mixed the results from animal and human studies) (5,6); moreover, our results considering the weighted means between secondary outcomes and the follow-up period feature a new data not yet available in scientific literature on the focused theme, which may provide a better understanding of peri-implant pathology in these cases; finally, a comprehensive online search was conducted in seven online databases, including searches on grey literature, and is in accordance to the items listed on quality and risk of bias assessment tools for systematic reviews, which also raised concerns with previous systematic reviews on this theme.

Our results may have been influenced by the risk of bias of included studies, which were of unclear risk of bias and raised concerns specially regarding the blinding of participants, personnel, and assessors. This may be considered as a limitation of our study (12).

One must highlight that a few - yet important - flaws in primary studies were identified during the review process and, if corrected, could reduce their risk of bias, providing more comprehensive results and improving the overall body of evidence, such as: a) concerns identified on risk of bias assessment (random sequence generation, allocation concealment, and blinding of participants, personnel and assessors) can be corrected if authors of primary studies do not choose a study design where only two groups are treated (infected and non-infected), which makes unpracticable to generate a random sequence, to conceal the allocation, and to blind participants, personnel, and assessors. We suggest that, instead of two groups, authors perform studies with at least three groups, whereas it would comprise non-infected sites and different types of infected sites such as infections of endodontic or periodontal origins; b) if an implant fails, authors should specify which type of infection lead to its loss (in cases where an implant was placed into an infected site). This would provide material so a subgroup analysis can determine the risk of failure for each type of infection;

The findings from the present systematic review are of the utmost importance for clinical practice, since it shows that immediate dental implants in infected sites present a risk of failure that is considerably higher than in non-infected sites, which corroborates to the statement that alveolar infection presents itself as an important risk factor for implant failure, which up to this point was not yet statistically proven in previous studies. Hence, these findings can be used to reduce the loss of costs, time and, most importantly, can preserve patients’ health.

Therefore, this systematic review and meta-analysis concluded the following:

- Immediate dental implants placed into infected sites is less effective than immediate dental implants placed into non-infected sites;

- There was a statistically significant higher risk of failure of immediate dental implants 

 placed in infected sites than immediate dental implants placed into non-infected sites;

- Peri-implant outcomes were not statistically affected in this intervention; however, there was an indication that peri-implant diseases may be more present around immediate dental implants placed into infected sites.
